# A Call to Expand the Scope of Digital Phenotyping

**DOI:** 10.2196/39546

**Published:** 2023-03-14

**Authors:** Christopher De Boer, Hassan Ghomrawi, Suhail Zeineddin, Samuel Linton, Soyang Kwon, Fizan Abdullah

**Affiliations:** 1 Division of Pediatric Surgery, Department of Surgery Ann & Robert H Lurie Children’s Hospital of Chicago Northwestern University Feinberg School of Medicine Chicago, IL United States; 2 Division of Rheumatology Department of Medicine Northwestern University Feinberg School of Medicine Chicago, IL United States; 3 Department of Pediatrics Ann & Robert H Lurie Children’s Hospital of Chicago Northwestern University Feinberg School of Medicine Chicago, IL United States; 4 The Smith Child Health Research Program Ann & Robert H Lurie Children's Hospital of Chicago Chicago, IL United States

**Keywords:** digital phenotyping, wearables, digital health, data collection, real-time, data, digital devices, smartphones, phenotype, quantification, phenotyping, wearable devices, tracking, monitoring, clinical data, applcaition, implementation

## Abstract

Digital phenotyping refers to near–real-time data collection from personal digital devices, particularly smartphones, to better quantify the human phenotype. Methodology using smartphones is often considered the gold standard by many for passive data collection within the field of digital phenotyping, which limits its applications mainly to adults or adolescents who use smartphones. However, other technologies, such as wearable devices, have evolved considerably in recent years to provide similar or better quality passive physiologic data of clinical relevance, thus expanding the potential of digital phenotyping applications to other patient populations. In this perspective, we argue for the continued expansion of digital phenotyping to include other potential gold standards in addition to smartphones and provide examples of currently excluded technologies and populations who may uniquely benefit from this technology.

## Introduction

Digital phenotyping is defined by Onnela and colleagues [1] as the “moment-by-moment quantification of the individual-level human phenotype in situ using data from personal digital devices, in particular smartphones” [2]. Digital phenotyping includes the key components of *continuous* data collection, on an *individual* level, captured in a *passive* way without the imposition of additional data collection tools. It provides new objective biomarkers for disease manifestations, previously reliant on subjective or self-reported data. This technical advancement has garnered substantial research interest due to both the hypothetical and realized promises of new *objective* ways of understanding and measuring diseases. For example, one study demonstrated that GPS data–based mobility and location anomalies gathered from a smartphone were associated with schizophrenia relapse, offering a new digital footprint of the disease [3].

Originating primarily in psychiatry, digital phenotyping addresses disease states that currently have no objective measurable biomarker, a quandary known as a *phenotyping problem*. For example, early studies demonstrated that smartphone data could be useful in identifying adolescents at risk for depression, bipolar disorder, and future stress [4-6]. Additional fields have also begun adopting this approach. For instance, others have used digital phenotyping to define recovery in surgical patients and monitor for signs of respiratory depression to potentially prevent opioid overdose [7-9]. With the ever-expanding wealth of digital health information available, digital phenotyping will become an inevitable part of medicine.

Despite these advances, there remains controversy over the optimal way (or gold standard) to collect data for digital phenotyping. The initial definition of digital phenotyping by Onnela and colleagues [1] has undoubtedly provided a new and necessary framework for understanding diseases. However, despite the aforementioned advances, it has been repeatedly argued that digital phenotyping should be used primarily via smartphones due to concerns about the availability, reliability, and data quality of other devices [1,10,11]. For example, it has been stated that smartphones are “ideally-suited” for digital phenotyping and represent “the *only* approach that makes it possible to aggregate data across studies and investigate aspects of behavior.” Other means of data collection, such as wearable devices, are considered to have “problematic” drawbacks such as attrition and data quality [11].

A smartphone-only approach to digital phenotyping excludes populations that cannot use or access a smartphone, such as young children, older adults who have vision impairments or have lost the ability to perform activities of daily living, and those with cognitive/behavioral barriers. In addition, smartphones can reliably provide GPS and actimetry data, but other vital signs, such as heart rate, temperature, and oxygen saturation, cannot be reliably obtained without a sensor placed on the core or wrist of the individual’s body. In addition, in today’s rapidly evolving technology landscape, the availability, reliability, and data quality of other devices have been significantly improved to address previous barriers that limited their inclusion in digital phenotyping. With such capabilities, we argue that there are significant opportunities to continue expanding the gold standard devices for collecting data used for digital phenotyping beyond smartphones and improving our understanding of human diseases. The purpose of this paper is to support the expansion of the definition and scope of digital phenotyping to include additional technologies and populations. While the inclusion of other devices, such as wearables, for digital phenotyping is not new, we argue for the widespread acceptance and adoption of these devices as another *needed* means for data collection in an effort to maximize the utility of digital phenotyping for medical care.

Of note, when considering digital phenotyping, this viewpoint recognizes the subtle differences between the terms “digital phenotype” introduced by Jain et al [12] in 2015 and “digital phenotyping” introduced by Onnela et al [1] in 2016. The former refers to the composition of disease-specific digital data and recognizes the range of devices from which it comes. The latter, however, refers to a field of science with a specific methodology that is wary of devices outside of smartphones. This viewpoint primarily refers to the latter, which is the specific methodology of carrying out digital phenotyping, although the former is affected as well. We also recognize more recent efforts to define the philosophical and epistemological underpinnings of digital phenotyping, which also expands original definitions. We seek to build on those by providing concrete examples of how an expanded definition of digital phenotyping plays out in real-world clinical applications [13].

## Limiting Digital Phenotyping to Smartphones Excludes Other Advanced Technologies for Capturing Physiologic Data

Smartphones have developed significantly and become ubiquitous in the last decade. However, other forms of smart devices, such as wearables, have also improved considerably in their ability to reliably collect and transmit passively collected physiologic data. Wearable sensors, such as accelerometers and heart rate monitors in the Fitbit or Apple Watch, present an incredible opportunity for the integration of passively collected digital information for digital phenotyping. These devices are rapidly advancing with applications ranging from traditional wrist-worn devices to patches, headbands, vests, glasses, and even implantable sensors within medical devices [14]. An advantage of these wearables is their direct contact with skin, which affords an opportunity for the collection of physiologic data, such as temperature and heart rate, which are not usually available through smartphones. A systematic review examining the validity of consumer wearable data found a “high” intra- and interdevice correlation of step counts in both laboratory and field studies and concluded that step counts from wearables were accurate with acceptable rates of error [15]. Heart rates obtained from consumer-grade wearables have also shown accuracy compared with standard electrocardiograms, with mean agreement rates as high as 95% [16]. With such capabilities, wearable sensors may have arguably *more* passive data collection capabilities in select scenarios than smartphones alone (their Bluetooth technology is often still needed for wearable data collection) because they can be used secondarily by a user who does not need to primarily manage the technical aspects (eg, a phone bill or account logins) of the device.

However, several concerns have been raised regarding the use of wearables for digital phenotyping [14]. First, use of these wearable sensors is growing at an exponential rate, and the number of globally connected devices is estimated to reach over 1 billion devices by 2022, a doubling of the number of devices in just 3 years [17]. If this rate of growth continues, these devices will be as ubiquitous as smartphones within a decade. Second, while older studies reported limited compliance with wearables [18], newer studies cite high compliance, satisfaction, and wear times in certain populations with adequate education and follow-up [19-21]. Third, one may also argue that adding devices represent an imposition that is unnatural and not passive, which could bias results. However, the rise of wearables means that they are likely a facet of our future digital lives (for health care or otherwise) that ought to be leveraged for digital phenotyping. In fact, studies are already using this by creating free and open enrollment into studies for the general public who already own and use wearables regularly to identify diseases and measure outcomes (eg, COVID-19) [22]. Finally, in addition to availability and reliability, the clinical incorporation of data from wearable sensors has improved considerably. According to a recent National Institutes of Health working group, wearables can be successfully incorporated into clinical practice when “key principles” identified by the group are followed [23]. This group further concluded that wearables “herald a new era in health care delivery with the potential to transform many aspects of clinical care.”

With this versatility, wearables offer an improvement in both the range and quality of data obtained compared to smartphones. The value of these additional data cannot be underestimated, and they have already been shown to be integral to the objective, remote identification of disease, and solution to a certain type of the aforementioned *phenotyping problem*. One needs to look no further than the current COVID-19 pandemic to see how wearable sensors proved useful in identifying signs of the disease reflected in heart rate, step counts, and sleep, up to multiple days prior to when patients would typically show symptoms [24,25]. In our studies, we have shown that step counts and heart rates from a wearable can be used to better understand surgical recovery and that this data can influence clinical decision-making [20,26].

These technologies are already advancing faster than the health care systems that are beginning to adopt them. Because of the rapidly changing technological landscape, new devices and technologies ought to be constantly evaluated for their utility in digital phenotyping. Without this, the definition and methodology of digital phenotyping will miss an opportunity to adapt along with evolving technologies and the wealth of information they contain.

## Limiting Digital Phenotyping to Smartphones Exclude Populations That Cannot Use or Access a Smartphone

A smartphone-only approach to digital phenotyping excludes populations that cannot use or access a smartphone on their own, such as young children, older adults who have vision impairments or have lost the ability to perform activities of daily living, and those with cognitive/behavioral barriers. These populations represent a significant missing component of more narrow applications of digital phenotyping that rely solely on smartphone use. Although adolescents are increasingly using smartphones (over 50% have a cell phone by the age of 11 in the United States [27]), there will always be age and cognition limits on the ability to use a smartphone. Just 61% of individuals older than the age of 65 years own a smartphone (compared to >90% in other age categories), with barriers to ownership in this age group cited as financial limitations, vision impairment, and lack of interest/knowledge in learning how to use the device [27].

In fact, proactive inclusion of these populations may have an even greater impact as these individuals are often the most vulnerable. For example, one study demonstrated that wearable sensors placed on infants demonstrated motion complexities that may predict the onset of autism spectrum disorder later in life [28], and another demonstrated gait characteristics that could predict an increased risk of a fall in older adults [29]. Beyond this, there may be many applications that could be truly transformative. For example, the digital phenotyping of pain in an irritable infant or of early stages of infection in a nursing home patient with dementia would be transformative for patient care and patient outcomes, providing an *objective* tool for anxious caregivers and health care providers in this scenario. Without a wearable, the collection of digital data from these populations who cannot use a smartphone on their own would not be possible. Exclusion of these populations not only limits the generalizability of digital phenotyping but also misses an incredible opportunity to assist specific populations of patients who may benefit from digital phenotyping the most.

## Toward a Broader Scope of Digital Phenotyping

**Figure 1:**
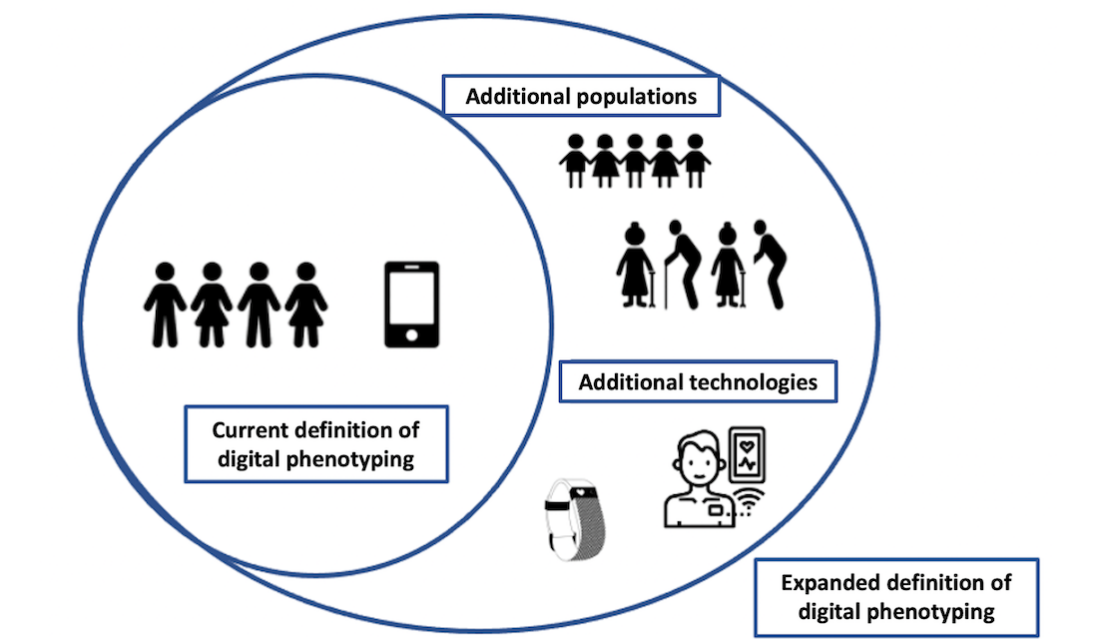
Conceptual framework for understanding an expanded definition of digital phenotyping that demonstrates that the foundation of digital phenotyping is rooted in a passive, smartphone-only approach but has the potential for an expanded definition that includes additional devices and populations.

To address the limitations of a focus on digital phenotyping on smartphones as the only gold standard, we propose an alternative framework where there are multiple gold standards for digital phenotyping in line with the diverse range of robust technologies that are currently available on the market. This in turn allows for better, more accurate digital phenotyping of certain diseases, and the inclusion of important missed populations (Figure 1). In fact, the literature review reveals that this idea is not entirely novel, although its widespread adoption would be, and much work is already being done, whether explicitly stated as digital phenotyping or not, that expands far beyond the use of smartphones.

For example, studies have combined smartphone and wearable data from wrist-worn or finger-worn devices to find associations between physical activity and self-reported feelings of loneliness, identify depression, and generate risk algorithms for suicidality [30-33]. Other studies have further expanded the types of data used, such as the incorporation of an accelerometer, gyroscope, compass, location, microphone, phone state indication, light, temperature, and barometer data from a smartwatch to describe emotional states [34]. Other fields, such as oncology and palliative care, have also begun to use wearables to track outcomes, offering new digital biomarkers of disease [35,36]. In addition to clinical applications of digital phenotyping, the ethical, philosophical, and epistemological aspects of digital phenotyping are now being described to better support the advancement of the field, including addressing topics such as data equity, data safety, and device reliability [37].

In addition to the more recent literature just discussed, a review of the origins of the definition of the phenotype and the process of phenotyping is helpful for supporting an expanded scope of digital phenotyping [13,38]. The phenotype is traditionally thought of in two ways: (1) in its original definition, it refers to the observable traits of an organism’s genetics; and (2) it also refers to the *extended phenotype*, as defined by Richard Dawkins [39] in 1978, which recognizes the effects of an organism’s genetics and corresponding phenotype on its surroundings. For example, beaver dams or beehives are expressions of that organism’s genetic code that alter the environment and, in turn, affect the populations of that environment. Digital phenotyping data from humans is analogous to beaver dams and beehives (eg, a beaver dam affects all the organisms in that environment, just as the digital data upon which clinical decisions will be based will in turn be generalized to the human population).

With these concepts in mind, the process of digital phenotyping ought to be as broad as possible without affecting the integrity of the process itself. The digital data that informs digital phenotyping will inform health care decisions and will subsequently affect people who may not have even provided that digital information initially. For this reason, it becomes paramount that novel digital footprints derived from digital phenotyping be based on as diverse digital information as is available. This means that while a completely passive process of digital phenotyping is certainly preferred, it only applies to those who can own and manage a device; have reliable Bluetooth and network technology; can consent to studies on their own; and, if needed, respond to surveys. On the other hand, some individuals will need devices provided to them, and, in some cases, devices in addition to smartphones may be preferred for the reasons explained above. Additionally, some populations may require additional resources and support to ensure data collection.

Smartphones are certainly the foundation for digital phenotyping and are, in fact, often necessary for the function of other devices via associated apps and Bluetooth technology. However, expanding the scope of digital phenotyping to include additional technologies and populations, which at times require active involvement by researchers, will allow for the unprecedented collection of digital data that is diverse, robust, and inclusive. Philosophically, this expansion respects the original definitions of the phenotype, and ethically, it ensures broader inclusivity of individuals in the process of digital phenotyping and the generalizability of the subsequent findings.

## Additional Considerations

As technologies advance and evolve, applications of digital phenotyping can also advance, evolve, and be tailored to specific populations. As mentioned above, this approach leads to a more inclusive and equitable expansion of digital phenotyping. However, additional considerations are needed beyond the technology itself.

Individuals affected by the digital divide ought to be considered in the future of digital phenotyping as well. This is a barrier that exists regardless of what type of technology is used. A quarter of individuals in the United States who earn less than US $30,000 do not own a smartphone or have the reliable, constant high-speed network connections necessary for data collection, and this access gap has persisted over time [27]. The inclusion of this population will not be solved with additional devices that are also reliant on smartphones and reliable networks themselves. Financial barriers will still exist even with the inclusion of wearables, and may even exacerbate disparities if not delivered equitably. Insurance coverage or provision of these devices (smartphones and wearables), free-of-charge to the patient, if necessary, not only addresses an issue of the ethical principle of justice in access to the future of digital health but also leads to opportunities for more robust understandings of disease based on an incredible diversity of digital information that is currently unobtainable given the known effects of social determinants of health. While we commend Onnela and colleagues [1] for examining the issue of socioeconomic disparities in the process of digital phenotyping, their conclusion that data can be reliably collected via smartphones across diverse populations was based on a small sample size of participants that were nearly all college students and nurses, which is not generalizable [40]. A more comprehensive understanding of the requirements of diverse data collection is needed and ought to be addressed in the future of digital phenotyping.

Funding for an expanded definition of digital phenotyping that includes additional devices is certainly a concern, but solutions are already being proposed or in place for a free-of-charge or reduced-charge provision of devices. For example, the Centers for Medicare and Medicaid Services has instituted Current Procedural Terminology codes for reimbursement of not only remote patient monitoring devices but also the clinical monitoring of their data under Medicare, with an anticipated expansion to Medicaid and private insurers as well [41]. A more balanced approach to digital phenotyping recognizes that there will always be a subset of individuals who will need devices or additional resources proactively provided to them lest they be excluded. This bias could severely threaten the validity and generalizability of findings based on digital phenotyping, as well as exacerbate socioeconomic-related health disparities.

Finally, one cannot ignore the fact that nearly all wearables rely on the coexisting ownership of a smartphone by the patient or their caretaker due to the cloud and Bluetooth capabilities for data storage and transmission. We are not suggesting that wearables or other devices replace smartphones in the future of digital phenotyping, as they are necessary, but rather that a more proactive provision of devices may be needed to ensure the inclusion of all.

## Examples of Expanded Applications of Digital Phenotyping: Advantages of Nonsmartphone Devices

Given the arguments, advantages, and barriers from above, the following represent examples where additional technologies and populations benefit from digital phenotyping and would be entirely excluded if a smartphone-only approach was taken. In the authors’ own experience and published results, wearables can address research quandaries that smartphones alone cannot, particularly in the care of children. In the case of surgical recovery, the physiological process of returning to homeostasis after the insult of surgery, wearables provide novel digital biomarkers of recovery where objective measurements are absent, particularly after discharge from the hospital. Defining this process in a measurable way is important for ensuring adequate recovery or identifying complications. In children, this is particularly important as they are often unable to adequately describe their symptoms and rely on proxies and parent reporting, which have been shown to be subjective and lead to suboptimal pain medication administration and unnecessary emergency room visits during postsurgical care [42-44]. Young children, who do not have the capacity to operate a smartphone, uniquely benefit from a wearable that can collect digital information that otherwise would have no means of being collected. This wearable data have shown that unique trajectories of recovery can be defined from physical activity data to more objectively describe surgical recovery after an appendectomy, offering a new objective biomarker of surgical recovery in children [20]. Segmental regression modeling of step counts by postoperative day demonstrated a rise and then a plateau, an indication of recovery. Further, this data, along with heart rate, can influence clinical decision-making [26].

Numerous other examples exist where nonsmartphone devices provide an advantage. For example, accelerometers and sensors on the sole, hip, and head can be used to create novel digital biomarkers of gait characteristics predictive of falls or of movement abnormalities for monitoring Parkinson disease [29,45]. Not only can wearables be placed on various locations on the body for novel metrics but they can also be worn passively by an older individual who may not be able to operate a smartphone, relying on Bluetooth aspects of data collection via the smartphone managed by a caregiver. Wearables also provide an advantage for populations in low-resource settings or those isolated from care. The expanded range of metrics available in wearables but not smartphones, such as wrist-based photoplethysmogram heart rate and pulse oximetry, or temperature from a wearable body patch, have replaced bedside monitors and temperature devices in patients isolated from care by providing metrics like clinically validated heart rate variability in an intensive care unit in Vietnam [46] and novel predictive algorithms for decompensation from COVID-19 in patients in the outpatient setting that outperformed the standard of care [47]. The addition of wearables in these scenarios not only incorporates entire populations that would otherwise be excluded but also creates an opportunity for additional types of digital information and metrics not captured by a smartphone. It must be reemphasized that wearables in these examples are not meant to *replace* smartphones, but rather serve as adjuncts that leverage existing smartphone availability and Bluetooth technology.

### Conclusions

The current scope of digital phenotyping is an invaluable first step toward leveraging digital information for new understandings of disease and ultimately improving health outcomes. An expansion of this scope to include other evolving technologies and populations that may, at times, require a more proactive approach will only strengthen digital phenotyping in the future. The incorporation of the wealth of digital information across numerous populations and technologies into digital phenotyping is possible and is already being done while still respecting key principles of original definitions. With this approach, the bounds of digital phenotyping are only as limited as the innovation and creativity of those who implement it.
